# Profiles and factors associated with schizophrenia in eastern Ethiopia: A matched case-control study

**DOI:** 10.3389/fpsyt.2022.1016005

**Published:** 2022-10-13

**Authors:** Fethia Mohammed, Biftu Geda, Tesfaye Assebe Yadeta, Yadeta Dessie

**Affiliations:** ^1^Department of Psychiatry, School of Nursing and Midwifery, College of Health and Medical Sciences, Haramaya University, Harar, Ethiopia; ^2^Department of Nursing, School of Health Sciences, Madda Walabu University, Shashamane, Ethiopia; ^3^School of Nursing and Midwifery, College of Health and Medical Sciences, Haramaya University, Harar, Ethiopia; ^4^School of Public Health, College of Health and Medical Sciences, Haramaya University, Harar, Ethiopia

**Keywords:** schizophrenia, mental illness, substance use, Harar, Ethiopia, factors

## Abstract

**Background:**

Despite its strong hereditary and genetic connections, there are other factors reported to be linked to schizophrenia, but not well studied in eastern Ethiopia.

**Objective:**

This study was aimed to investigating the potential profiles and factors associated with schizophrenia in eastern Ethiopia.

**Materials and methods:**

A matched case-control study was conducted in two public hospitals from December 1, 2021, to January 30, 2022. Cases were patients with schizophrenia who visited the hospitals, and controls were healthy individuals without any mental illness who visited the same hospitals. A questionnaire was used to collect the data. Cases and controls were matched using age and sex. STATA-14 was used for analysis. A conditional logistic regression with an adjusted odds ratio (AOR) and a 95% confidence interval (CI) was applied to identify the determinants. *P*-values of <0.05 were used to build the final model as a measure of statistical significance.

**Results:**

The mean age of the study participants group was 28.6 (±8.44) years, mean age for cases was 28.7(±8.5) ranging from 18 to 56 years and the mean age for the controls was 28.4 (±8.5), ranging from 18 to 60 years. About 181 (83.03%) of the participants were male. The odds of having schizophrenia was about 12.2 times higher among participants with family history of mental illness (AOR: 12.21; 95% CI: 4.83–30.00). The odds of having schizophrenia was 4.5 times higher among polysubstance users (AOR: 4.45; 95% CI: 1.28–5.45) and 2.8 times higher among khat consumers (AOR: 2.82; 95% CI: 1.23–6.45) compared to their counterparts.

**Conclusion:**

Our findings show that genetic risk factors as well as some modifiable behaviors are associated to schizophrenia in eastern Ethiopia. At all levels, special attention should be given to those who are at risk.

## Introduction

Schizophrenia is a serious, chronic mental illness (1) influenced by and happens due to a number of genetic and environmental factors (2). Evidence have shown that relatives of patients with schizophrenia have a 5–10 times higher risk of developing schizophrenia (3), and those with a positive family history of mental illness are more likely to develop the problem at a younger age (4). Moreover, many environmental factors have been identified to contribute to the risk of developing schizophrenia at various times, especially during development stages (prenatal life, perinatal life, adolescence, and adulthood) (5). Such environmental factors have additive or cumulative effects, which may be linked and/or share causal factors (6). Overall, the likelihood of developing schizophrenia is ascribed to interactions between genetic and environmental factors (6). Evidence has shown that those who experience malnutrition, stress during pregnancy, perinatal abnormalities, and social problems (7), stressful situations, vitamin D insufficiency, childhood maltreatment and abuse are reported to be linked with developing schizophrenia (8, 9).

Moreover, psychoactive substance use is so often linked to schizophrenia (10), and particularly, there is a high relationship between cannabis use and this disease (11). A study suggests that psychoactive substances (such as amphetamines) can induce schizophrenia (12). Those who begin using a psychoactive substance such at a younger age and who use it at high potency or use it more frequently have a stronger tendency to develop schizophrenia (13, 14). The continued use of such substances after the initial episode is connected to a worse prognosis, higher relapse rates, longer hospitalizations, and severe positive symptoms (15). In other words, the age at which this substance use begins appears to be related to the age at which the problem reveals itself (11).

People with schizophrenia are more likely to have dysphoric feelings, which make them more likely to take psychoactive substances (16). According to the available data, people with schizophrenia use narcotics to mask the uncomfortable feelings just before the addiction process takes over (17, 18). The use of psychoactive substances by people with schizophrenia has been found to have a variety of negative effects in the course of the illness, such as poor adherence to their medication, poor prognosis, and a higher rate of emergency services utilization, which, in the end, results in higher healthcare costs (19). A family history of mental illness, substance use, especially cannabis and amphetamine use, and other environmental factors, such as unemployment, social isolation, and housing insecurity, all contribute to the onset of schizophrenia. As a result, these factors increasingly highlight the diverse and multidimensional nature of schizophrenia (20). In this respect, there are very limited empirical studies in Ethiopia, and more importantly, in eastern Ethiopia. Therefore, the objective of this study was to assess the Profiles and factors associated with schizophrenia in eastern Ethiopia.

## Materials and methods

### Study setting and design

This study was conducted from December 1, 2021, to January 30, 2022, at Hiwot Fana Comprehensive Specialized University Hospital and DilChora Referral Hospital. Hiwot Fana Comprehensive Specialized University Hospital is located in Harar town. Harar is the capital city of Harari Regional State, located in eastern Ethiopia at a distance of 526 km from Addis Ababa. There are two public hospitals in the Harari Region (Hiwot Fana Specialized University Hospital and Jogul Regional Referral Hospital). The Hospital was established in 1941 and became a university specialized hospital in 2010. It provides service to more than 5.8 million people in its catchment area of eastern Ethiopia. DilChora Referral Hospital is located in Dire Dawa city administration, which is 515 km from Addis Ababa. It is the only referral hospital in the city and was established in 1960 GC. DilChora Referral Hospital, located in the northeast of the city, provides service for more than 45,000 people in the catchment area of eastern Ethiopia.

### Study design

A hospital-based matched case–control study design was used. The cases were primary patients with schizophrenia who were individually matched for age (±5 years) and sex with a healthy control (without any mental illness) from the two hospitals. Patients with schizophrenia over the age of 18 who were confirmed by a psychiatrist to have schizophrenia and were on antipsychotic medication for the previous three months were considered cases. The cases were selected when they visited the hospitals for follow-up and collected their antipsychotic medications. Their condition was stable and confirmed to be free of acute symptoms. In contrast, the control group consisted of healthy individuals (age greater than or equal to 18) who had never been diagnosed with any type of mental illness or had never had any other chronic medical or surgical illness but visited the hospitals within the same time frame as the cases. The age (±5 years) and gender of the case were matched to the corresponding control in order to check if he or she had the most similar matching conditions to the case.

### Data collection instrument and procedure

A structured questionnaire was used to collect data about the participants’ sociodemographic characteristics, clinical factors, and substance use. Involvement Screening Tests (ASSIST) was used to collect information about the participants’ substance use behavior (21).

Each control group was examined by the psychiatrist using a structured clinical interview for DSM-IV, which addresses each symptom domain and ensures an accurate and reliable psychiatric diagnosis. The psychiatrist confirms the absence of psychiatric symptoms (22).

### Data quality

The questionnaire was prepared in the English language and translated into local languages of the participants (Afaan Oromo and Amharic) so that the respondents understand the items clearly. Finally, the questionnaire was translated back to English version to ensure its consistency with the initial version.

Data collectors were two psychiatrists and two psychiatry nurses with B.Sc. degree and two supervisors with M.Sc. degree in integrated clinical and community mental health. The data collectors, along with the supervisors, were trained for 2 days regarding the data collection procedure. Data was collected through face-to-face interviews using structured and pre-tested questionnaires. The interviews were conducted in privet room of psychiatric clinic, the controls, participants were also interviewed on the same day by the same interviewer.

Moreover, to enhance the quality of the data collection tool and validity of the data, pre-test was conducted on 5% of the total sampled participants in Haramaya Hospital located in the Haramaya town, eastern Ethiopia. The results were used for the improvement of the questionnaire items. Supervisors and the principal investigator checked for completeness and consistency of the collected data. On a daily basis, supervisors and the principal investigator checked the collected data for completeness and consistency. Codes were given to completed questionnaires, and double data entry and verification were performed to avoid any errors during data entry.

### Measurements

The diagnosis of schizophrenia was based on DSM-5 and diagnostic label was given by the psychiatrists. Schizophrenia was measured by presence of two (or more) of the following symptoms: delusion, hallucinations, disorganized speech, behaviors with impaired social or occupational functions at least for the last 6 months (23).

### Statistical analysis

The collected data were checked, coded, paired with Id and entered into EpiData version 3.02 and exported to STATA 14 statistical software for analysis. Descriptive statistics were done using frequency tables. A conditional logistic regression model was used to account for the matched case–control study design. Bivariable analysis was done for computing unadjusted matched odds ratios (ORs) and their 95% confidence intervals (CIs). The level of significance was assessed with *p*-values < 0.02, entered into a multivariable conditional logistic regression model using group identifications to simultaneously examine their independent effects (adjusted ORs, AORs). The final model was obtained with *p*-values < 0.05.

### Ethical considerations

Ethical clearance was obtained from the Institutional Health Research Ethical Review Committee (IHRERC) of the College of Health and Medical Sciences, Haramaya University (Ref.No. IHRERC/255/2020). A copy of the ethical clearance letter was submitted to the hospital administration. A letter of cooperation was written to the respective hospital psychiatry departments. Consent to participate in the study was obtained from all the study participants before data collection. Each informed, voluntary participant had signed on the written consent form all of which was obtained by the researchers. In addition, informed, voluntary, written, and signed assent was obtained from the caretakers and parents of schizophrenia patients (case). All the study participants were well informed about their rights to withdraw from the study at any stage. Confidentiality of the information was assured.

## Results

This study included a total of 436 individuals, i.e., 218 cases and 218 controls. The overall mean age of study participants was 28.6 (SD ± 8.44) years old. The mean age for case group was 28.7(SD ± 8.5) whereas it was 28.4 (SD +8.5) for control group. A total of 137 (62.84%) of the controls and 142 (65.14%) of the cases were from urban. A total of 82 (37.61%) of cases were at age category of 18–24 years compared with 82 (37.61) controls; and, 85 (38.99%) of cases were within the age category of 25–34 years compared with 86 (39.45%) of controls. About two-third 137 (63%) of the cases were Muslim in religion compared to 138 (63.30%) controls and flowed by Orthodox Christians religion, followers which was, 62 (28.44%) cases and 57 (26.15%) among controls. About half, 102 (46.79%), of the case group and one third, 71(32.57%), of the control group had no formal education, and 104 (47.71%) of the cases and 78 (35.78%) of the controls were single. Thirty-four (15.60%) of the cases and 80 (36.70%) of the controls were employed in government offices (Table 1).

**TABLE 1:** Socio-demographic characteristics of the study participants at selected public hospitals in eastern Ethiopia from December 1, 2021 to January 30, 2022.

Characteristics	Categories	Case (*N* = 218)		Control (*N* = 218)	
		Frequency	%	Frequency	%
Gender	Male Female	181 37	83.03 16.97	181 37	83.03 16.97
Residence	Urban Rural	142 76	65.14 34.86	137 81	62.84 37.16
Age in years	18–24 25–34 >35	82 85 51	37.61 38.99 23.39	82 86 50	37.61 39.45 22.94
Marital status	Married Single Separated	32 104 82	14.68 47.71 37.61	84 78 56	38.53 35.78 25.69
Education	No formal education Primary level High school and above	102 67 49	46.69 30.73 22.48	71 68 79	32.57 31.13 36.24
Occupation	Employed Farmers Jobless Other	34 32 109 43	15.60 14.68 50.00 19.72	80 30 65 43	36.70 13.76 29.82 19.72

### Substance use among study participants

The results showed that 62.39% of the cases and 14.22% of the controls had history of lifetime khat use. Current khat consumers were 71 (32.57%) among the controls and as high as 131(60.09%) among the cases. Also, 7 (3.21%) of the controls had family problems related to their khat consumption. Family history of mental illness was reported to be 102 (46.79%) among the cases and 38 (17.43%) among the controls. Family history of substance use was found to be 115 (52.75%) among the cases and 9.17%, among the controls (see Table 2).

**TABLE 2:** Substance use characteristics of study participants at selected public Hospitals in eastern Ethiopia from December 1, 2021 to January 30, 2022.

Characteristics	Categories	Cases (*N* = 218)		Control (*N* = 218)	
		Frequency	%	Frequency	%
Lifetime tobacco use	Yes No	35 183	16.06 83.94	28 190	12.84 87.16
Current tobacco use	Yes No	68 150	31.19 68.81	44 174	20.18 79.82
Smock less tobacco use	Yes No	49 169	22.48 77.52	12 206	5.50 94.50
Alcohol consumption	Yes No	56 162	25.69 74.31	34 184	15.60 84.40
Lifetime khat use	Yes No	136 82	62.39 37.61	31 187	14.22 85.78
Current khat use	Yes No	131 87	60.09 39.91	71 147	32.57 67.43
Poly substance use	Yes No	54 164	24.77 75.23	17 201	7.80 92.20
Have problem with their family due to khat chewing habit	Yes No	32 182	14.68 85.32	7 211	3.21 96.79
Family history of mental health problem	No Yes	102 116	46.79 53.21	198 20	90.83 9.17
Family history of substance use	No Yes	103 115	47.25 52.75	180 38	82.57 17.43

### Factors associated with schizophrenia

The obtained final model has shown that the odds of having schizophrenia was about 3.11 times higher [AOR 3.11; 95% CI (1.30–7.42)] among those who did not have formal education compared to those who attended formal education. The odds of having schizophrenia was 6.15 times higher among singles (AOR: 6.15; 95% CI: 2.47–15.28) and 5.36 time higher among separated individuals (AOR: 5.36; 95% CI: 2.18–13.19) compared to those who were married and still kept their marriage. Furthermore, the odds of having schizophrenia was 4.60 times higher among the jobless (AOR: 4.60; 95% CI: 1.80–11.71) compared to the employed. Likewise, the odds of having schizophrenia was about 12.2 times higher among participants with positive family history of mental illness compared to their counterparts (AOR: 12.21; 95% CI: 4.83–0.84) and cases were found to be more likely among those participants with family history of substance use (AOR: 2.13; 95% CI: 1.07–4.29). The odds of having schizophrenia was 2.31 times higher among current tobacco users (AOR: 2.31; 95% CI: 1.08–4.91), 2.82 times higher among current khat users (AOR: 2.82; 95% CI: 1.23–6.45) and 4.45 times higher among polysubstance users (AOR; 4.45; 95% CI: 1.28–15.45) compared to their counterparts (see Table 3).

**TABLE 3:** Bivariable and Multivariable conditional logistic regression model showing risk factors of schizophrenia at selected public hospitals in eastern Ethiopia from December 1, 2021 to January 30, 2022.

Variables	Categories	Schizophrenia		COR (95%CI)	AOR (95%CI)
		Yes (%) case	No (%) control		
Gender	Male	181 (83.03)	181 (83.03)	1. (0.58–1.70)	1.35 (0.55–3.29)
	Female	37 (16.97)	37 (16.97)	1	1
Residence	Urban	142 (65.14)	137 (62.84)	1.12 (0.74–1.70)	1.07 (0.51–2.24)
	Rural	76 (34.86)	81 (37.16)	1	1
Educational status	No formal education	102 (46.79)	71 (32.57)	2.61 (1.56–4.37)*	3.11 (1.30–7.42)*
	Primary	67 (30.73)	68 (31.19)	1.69 (1.01–2.81)*	2.17 (0.81–5.81)
	Secondary and above	49 (22.48)	79 (36.24)	1	1
Marital status	Married	32 (14.68)	84 (38.53)	1	1
	Single	104 (47.71)	78 (35.78)	3.54 (2.06–6.07)*	6.15 (2.47–15.28)*
	Separated	82 (37.61)	56 (25.69)	3.67 (2.09–6.43)*	5.36 (2.18–13.19)*
Occupation	Employed	34 (15.60)	80 (36.70)	1	1
	Farmer	32 (14.68)	30 (13.76)	2.94 (1.36–6.36)*	2.27 (0.65–7.96)
	Jobless	109 (50.00)	65 (29.82)	4.31 (2.43–7.64)*	4.60 (1.80–11.71)*
	Other^a^	43 (19.72)	43 (19.72)	2.48 (1.25–4.93)	3.71 (1.23–11.15)*
Family history of mental illness	No	116 (53.21)	198 (90.83)	1	1
	Yes	102 (46.79)	20 (9.17)	7.75 (4.25–14.14)*	12.21 (4.83–30.84)*
Family history of substance use	No	103 (47.25)	180 (82.57)	1	1
	Yes	115 (52.75)	38 (17.43)	4.5 (2.84–7.14)*	2.13 (1.07–4.29)*
Current tobacco use	No	150 (68.81)	174 (79.82)	1	1
	Yes	68 (31.19)	44 (20.18)	1.69 (1.11–2.56)*	2.31 (1.08–4.91)*
Currently consuming khat	No	87 (39.91)	147 (67.43)	1	1
	Yes	131 (60.09)	71 (32.57)	3.72 (2.33–5.97)*	2.82 (1.23–6.45)*
Poly substance use	No	164 (75.23)	201 (92.20)	1	1
	Yes	54 (24.77)	17 (7.80)	3.71 (2.06–6.70)*	4.45 (1.28–15.45)*

COR, crude odds ratio; AOR, adjusted odds ratio.

*P < 0.05.

## Discussion

To iterate, this study aimed at assessing the potential Profiles and factors associated with schizophrenia in eastern Ethiopia using a matched case-control study. Conditional logistic regression analysis was used to examine the risk factors for schizophrenia.

Family history of mental illness, history of khat consumption, cigarettes smoking and use of other polysubstance were found to be strongly associated with schizophrenia. Moreover, educational status and type of occupation were significantly associated with schizophrenia. The chances of having schizophrenia were higher among those with no formal educations, those with a positive family history of mental illness and family history of substance use as well as among the single and the unemployed individuals. Khat was the most commonly used substance in the study area, with more than half of the patients having consumed khat at some point in their lives and 60.09% were currently consuming khat while having family problems as a result of consuming it.

In this study, having no formal education was associated with schizophrenia, This finding support’s the earlier study (24). This might indicate that schizophrenia is linked to deficits in cognitive function as well as lower educational achievement because, individuals with schizophrenia are vulnerable to discrimination in a variety of settings, which can result in job loss and difficulty in finding new job. Furthermore, being disadvantaged in formal education, experiencing unemployment and schizophrenia are all part of a vicious cycle which frequently result in poor academic performance and unemployment (25–27),or low educational level and Poverty are inextricably linked, that patients with schizophrenia are more likely to be poor than healthy people, those with a lower level of education and poor may have less access to supportive services than those with a higher level of education, ultimately leading to a poor prognosis, poverty, as a socioeconomic selection process mediator, may have had a role in the apparent association between inadequate education and schizophrenia (24, 28).

The result of this study showed that the odds of schizophrenia were higher among those who are single and separated compared with married individuals. This means those who are single or separated experience the highest levels of stress (29). They are often socially isolated and yet bear additional burden in terms of economic and emotional well-being, which leads to the stress. Those in marriage may have a better psychological adaptation and physical wellbeing than those who are separated or have never been married. In other words, marriage is strongly linked to a better outcome in patients with schizophrenia because they receive the necessary assistance from their spouses and relatives, which might result in an overall better mental and physical health status than those who are never married or are single. As a result, married patients are able to look after themselves and cope with stress (29, 30).

The present finding shows that family history with mental illness was found to be associated with schizophrenia. This might be due to the fact that gene–environment interactions play a role in the etiology of schizophrenia (31). It might also be due to shared risk of genetic markers, environmental exposures or a combination of these factors with family history of mental illness (32). Previous studies have also shown that family history of mental illness have biological/hereditary effects and that other psychiatric problems in the family could indicate similar etiological factors in the development of the problems (33, 34).

Moreover, the results of this study revealed that a family history of substance use was higher among cases than control group. This is because family members share similar characteristics that could be risk factors for substance abuse. Substance dependence can be inherited from parents due to genetic and/or environmental factors and there is an environmental factor that can mediate substance dependence. Siblings of substance-abusing families may have more access to substances and thus have a higher risk of developing substance misuse (35). Furthermore, the findings of the present study indicate that substance users were 2.82 times more likely to have schizophrenia compared to non-users (AOR: 2.82; 95% CI: 1.23–6.45). Several factors have been identified as contributing factors to substance use among people with schizophrenia. People use substances because (they believe) these might relieve or help to cope with psychotic symptoms, relieve unpleasant emotional feelings, reduce antipsychotic medication side effects, increase pleasure, make feel more energetic or good, or improve mental concentration (e.g., during reading) and/or help for sociocultural purposes (16, 36). In addition, the findings of this study also show that substance use is significantly higher among single (unmarried) participants, This finding supports previous study report (33). The most likely cause is lack of social support that may lead to “self-medication” with a psychoactive substance which (is believed) gives relief from negative symptom and medication side effects as well (30).

Our results demonstrate a high rate of substance use among patients with schizophrenia, which is consistent with other studies (37).

The most commonly used substance was khat followed by nicotine. Substance use was 31.19% among cases (schizophrenia patients) and 20.18% among controls (healthy individuals), of which 60.1% of the cases were current khat consumers. The type of substance used and the patterns of use in people with schizophrenia indicate that the availability of different types of substances determine which substances are commonly used, rather than their effects (38). This might be because of socially constructed cognitive components related to the substance, for instance perceptions, demands and other motivating factors for substance use contribute to continued substance use in schizophrenia (39). Possible reasons include easy accessibility, low cost, and cultural acceptance of, for instance khat, in the study area (34, 40, 41).

What’s more, according to the present findings, substance use is higher in males than females, in younger than elderly populations. This finding is in line with the previous researchers (33, 42, 43). In other words, gender and age are important factors that influence substance use (44). Cultural and biological phenomenon with gender-specific developmental issues may also lead to substance use (39). Men and younger people are more susceptible to substance use than women and elderly population because the former group want to participate in hazardous behaviors like experimenting with substances use, yet female possibly begin self-medicating with substances to relieve the symptoms (45).

Tobacco use was also found to be significantly higher in males with schizophrenia than in their control counterparts this findings support other study regarding use of tobacco among patients with schizophrenia (46). It was thought that patients with schizophrenia smoke tobacco to improve hepatic clearance and restore the dopamine blockade caused by antipsychotic medications (43), thus reducing their side effects (17, 18). Smoking cigarettes may perpetuate among patients with schizophrenia to mask uncomfortable feelings (47), because it relieves psychiatric symptoms, particularly negative and cognitive one. The findings of the present study supports the previous studies in terms of patients’ substance use (43, 48).In this study more than half of the schizophrenia patients consume khat possibly because they use psychoactive substances to “self-medicate” in order to “feel good,” “get high,” or “relax,” and socialize with their peers (49). The fact that khat is widely available in lower cost and peer influence may commonly expose them to consuming it (7, 49, 50). Family attitude toward khat consumption may also be a risk factor for khat consuming (51, 52).

Finally, the results of this study indicate that cases with schizophrenia are more likely to use poly-substances than the controls. This could be because of availability, affordability, ease of access, cultural context, and social networks that influence the substance use (49). In the study area, the neurobiological basis (49, 53), more flexible social attitudes toward these poly-substances may facilitates the use of these substances (54). In addition, patients with schizophrenia may use poly-substances as a form of self-medication, which facilitates in the reduction of negative symptoms and the improvement of cognitive abilities. It could also be due to behavioral concordance of using one type of substance with another or the illness may require the use of multiple substances at once (53).

### Strength of the study

As to the knowledge of the researchers, this study is possibly the only matched case-control study which attempted to assess the Profiles and factors associated with schizophrenia in the context of eastern Ethiopia. It is believed that it provides useful finding for understanding risk factors of schizophrenia, which might also be transferable to the wider Ethiopian context.

The limitations of this study are that there is a risk of underestimation because stigma in Ethiopia restricts admittance to a positive family history. When interviewers administer questionnaires, people who use khat and other substances tend to under-report or deny their use. In addition, because the study was conducted in a hospital, it cannot be generalized to all patients in Ethiopia.

## Conclusion

Our findings reveal that genetic risk factors such as a family history of mental illness and substance use patterns are among the most significant risk factors for schizophrenia in eastern Ethiopia, khat chewing, unemployment and poly-substance use are among the key modifiable behaviors associated with schizophrenia. Knowing these factors can aid in the early detection and prevention of the condition. There must be interventions to address those modifiable behavioral variables. Especially, substance users and people with family history of mental illness need special attention at all possible interventions in community and clinical settings is mandatory.

## Data availability statement

The raw data supporting the conclusions of this article will be made available by the authors, without undue reservation.

## Ethics statement

Ethical clearance was obtained from the Institutional Health Research Ethical Review Committee (IHRERC) of the College of Health and Medical Sciences, Haramaya University (Ref.No. IHRERC/255/2020). The patients/participants provided their written informed consent to participate in this study.

## Author contributions

All authors have made a significant contribution to the conception the study, study design, data collection, data analysis, and interpretation of the results, took part in writing the manuscript, reviewed the draft, and finally agreed on the journal to which the article has to be published, read and approved the final draft of the manuscript and agreed to be accountable for all contents of the manuscript under any circumstances.
